# Assessing global, regional, national and sub–national capacity for public health research: a bibliometric analysis of the Web of Science^TM^ in 1996–2010

**DOI:** 10.7189/jogh.06.010504

**Published:** 2016-06

**Authors:** Anna Badenhorst, Parisa Mansoori, Kit Yee Chan

**Affiliations:** 1 Centre for Global Health Research and WHO Collaborating Centre for Population Health Research and Training, The Usher Institute for Population Health Sciences and Informatics, University of Edinburgh, Scotland, UK; 2 Nossal Institute for Global Health, University of Melbourne, Victoria, Australia

## Abstract

**Background:**

The past two decades have seen a large increase in investment in global public health research. There is a need for increased coordination and accountability, particularly in understanding where funding is being allocated and who has capacity to perform research. In this paper, we aim to assess global, regional, national and sub–national capacity for public health research and how it is changing over time in different parts of the world.

**Methods:**

To allow comparisons of regions, countries and universities/research institutes over time, we relied on Web of Science^TM^ database and used Hirsch (h) index based on 5–year–periods (h5). We defined articles relevant to public health research with 98% specificity using the combination of search terms relevant to public health, epidemiology or meta–analysis. Based on those selected papers, we computed h5 for each country of the world and their main universities/research institutes for these 5–year time periods: 1996–2000, 2001–2005 and 2006–2010. We computed h5 with a 3–year–window after each time period, to allow citations from more recent years to accumulate. Among the papers contributing to h5–core, we explored a topic/disease under investigation, “instrument” of health research used (eg, descriptive, discovery, development or delivery research); and universities/research institutes contributing to h5–core.

**Results:**

Globally, the majority of public health research has been conducted in North America and Europe, but other regions (particularly Eastern Mediterranean and South–East Asia) are showing greater improvement rate and are rapidly gaining capacity. Moreover, several African nations performed particularly well when their research output is adjusted by their gross domestic product (GDP). In the regions gaining capacity, universities are contributing more substantially to the h–core publications than other research institutions. In all regions of the world, the topics of articles in h–core are shifting from communicable to non–communicable diseases (NCDs). There is also a trend of reduction in “discovery” research and increase in “delivery” research.

**Conclusion:**

Funding agencies and research policy makers should recognise nations where public health research capacity is increasing. These countries are worthy of increased investment in order to further increase the production of high quality local research and continue to develop their research capacity. Similarly, universities that contribute substantially to national research capacity should be recognised and supported. Biomedical journals should also take notice to ensure equity in peer–review process and provide researchers from all countries an equal opportunity to publish high–quality research and reduce financial barriers to accessing these journals.

Investment in global public health research and development has seen a huge increase in recent years. Funding for health research increased from US$ 50 billion in 1993 to US$ 240 billion in 2009 [1], whilst financial contributions to international Development Assistance for Health (DAH) increased from US$ 5.6 billion to US$ 28.1 billion between 1990 and 2012 [1]. These substantial increases in funding have coincided with a “paradigm shift” from “*International Health*” to “*Global Health*”, which occurred over the past two decades. “*International Health*” had its focus on national public health efforts to assist poorer countries [2]. However, “*Global Health*” centres its attention on “*collaborative transnational research and action for promoting health for all*” [3]. This shift provoked recognition that collaborative global action was required to tackle new and evolving health issues, such as SARS, pandemic flu, Ebola, re–emergence of tuberculosis or increase in antibiotic resistance. Additional concerns were raised over the rapidly increasing burden of non–communicable diseases (NCDs) and the need to address health inequities within and between countries [4].

The landscape of global health changed, too, with the World Health Organization and specific countries no longer being seen as the only relevant actors in global health, and with hundreds of organisations now funding global health in an increasingly complex and fragmented manner [5,6]. Whilst the increase in available funding opens up new realms of possibility within global public health research, there is a demand for increased coordination. There were a number of attempts to track and monitor the funding for health research [1,7–10], yet their estimates are strikingly varied, revealing methodological challenges in categorising how the money is spent. To ensure that funding for global health research is being efficiently used, it is necessary not only to understand what is being supported, but also how the funding allocation relates to national and institutional capacity for global health research. Locations with improved capacity for research that are being under–utilised should be identified. As an example, it has been shown that the BRICS nations (Brazil, Russia, India, China and South Africa) made a considerable academic progress in the 21st century: between 2002 and 2007, India doubled the number of original health research papers they produced from 4494 to 9066 [11]; whilst Elsevier (2013) reported that these emerging nations, particularly China, were beginning to overpower the traditional stalwarts such as the UK and USA through the volume of research they are producing. However, it is not only the quantity but also the quality of research, which is improving [12]. To our knowledge, no comprehensive evaluation of the capacity for global public health research has been conducted and the changes in this capacity explored.

In trying to map the capacity, several tools may be utilised. Bibliometric tools allow an evaluation of research productivity, quality, visibility and/or impact at an individual to global level, and therefore can provide a measure of capacity for research. They present objective evidence to describe current research trends and development. The most used bibliometric tools, their advantages and limitations are outlined below. The aim of this study is to assess global capacity for public health research and progression of changes in this capacity over time. In order to achieve this aim, the following objectives must be met:

1. To develop a new scientometric approach, based on *h*–index, which allows an assessment of research characteristics of institutions, countries and regions and their comparison over time;

2. To perform a bibliometric analysis of global public health research based on *h*–index, which is calculated by the Web of Science^TM^;

3. To identify countries and Universities that are improving their capacity for public health research, and those that are stagnating or lagging behind;

4. To identify the research topics of interest within global public health, and their trends over time.

## METHODS

### Definition of geographic regions and countries included in this study

The countries within each region were defined using the six World Health Organisation's regions [13]. Two of the WHO regions were further subdivided, resulting in a total of 8 separate regions. This was done in order to allow a more comprehensive representation of LMIC and the BRICS nations. The additional regional groupings were created by further dividing the Americas and West Pacific WHO regions into Americas I and II, and West Pacific I and II [14]. A total of 193 countries were included in the analysis. The countries included are shown by region in **Online Supplementary Document(Online Supplementary Document)**. As the country list was taken from the WHO, disputed countries or territories were not analysed, including Kosovo and Taiwan. The countries that had merged, separated or changed their status or names between 1996 and 2010 were only analysed using their current name (in 2015). Wherever possible, countries with names that have different formats, spelling or abbreviations were identified and all formats of the name used in the search. Due to address restrictions on WoS, publication and citation data from Sudan and South Sudan was aggregated and presented as Sudan and considered in the Eastern Mediterranean Region (EMR). The UK was presented as a single statistical entity, combining England, Scotland, Wales and Northern Ireland.

### Definition of time periods

The h–indices, calculated by the Web of Science^TM^, were investigated over three time periods, each of five years: 1996–2000, 2001–2005 and 2006–2010. Five–year periods were chosen to reduce year–to–year stochastic variation within countries. To accommodate for the expected lag between publications and citations, a “citation window” of an additional 3 years following each 5–year period was allowed. This means that, eg, when calculating the h–index for the 5–year time period spanning between 1996–2000, publications with dates 1996–2000 were included, but all citations attributed to those publications in the period 1996–2003 were taken into account in calculation of h–index. This also attenuated the concern related to the temporal nature of the h–index, where older publications would have had a longer time period within which they would have attracted citations.

### Search of the literature

After considering the information obtained through the literature review using several available databases (eg, Scopus, Google Scholar and Web of Science), and examining the strengths and weaknesses of each database, Web of Science^TM^ (WoS) was chosen as the database used for this bibliometric analysis. The WoS “Core Collection” was used to ensure that only the publications in the journals with regularly assessed quality are considered.

Given that “public health” is not available as a specific category of articles within WoS, and given that alternative pre–defined categories available in the WoS have serious limitations, it was necessary to devise a search strategy that would efficiently identify public health research to enable an assessment of global, regional, national and sub–national capacity for such research. The search strategy needed to allow an evaluation that would be fair to all countries and allow their meaningful comparison. Public health research can include a multitude of topics, but we chose three search terms as highly specific “indicators” of public health research, as opposed to other types of health research. Those were “epidemiology”, “public health” or “meta–analysis”. The first two are clear indicators of public health research, whilst meta–analyses are increasingly being performed in response to a growing need to generate evidence for health policy. Although we could have arguably included the term “systematic reviews”, we felt that the more rigorous methodology that underlies a meta–analysis process would be a better indicator of research capacity. The search was automated so that all the papers that had any of the words “public health OR epidemiology OR meta–analysis” anywhere in the article were identified by Web of Science^TM^. There were no follow–up steps to this search and all subsequent analyses were then performed on the identified sample of studies.

We validated this approach through studying all 2654 articles that contributed to any of the regional h–indices throughout any of the three time periods (and formed a sub–sample of about 1% of all retrieved articles). One researcher (AB) read the title and the abstract to verify if the article was indeed related to public health. Among those, 58 articles were not related to public health topics, and there were no ambiguities – most of them were meta–analyses related to environmental sciences. This meant that our chosen approach showed about 98% specificity in finding the articles in h–core that are relevant to public health. Whereas the sensitivity of our approach would be very difficult to estimate, the high level of specificity was very encouraging.

### Categorisation of papers by type of research and topics of research

To analyse the types of research and the topics of interest that were studied globally over the three time periods, the abstract of each publication contributing to the h–core was reviewed and the publication was categorized using a number of criteria. In terms of topics, papers were characterized as being mainly related to the study of non–communicable diseases (NCDs), infectious diseases (ID), other diseases, or predominantly methodological papers. According to instruments (domains) of the research that were used, a conceptual framework proposed by Rudan et al. was used [15], with the 4 categories and the criteria for categorization shown in **Table 1**.

**Table 1:** Research instruments (domains) in global public health research*

**Research domain**	**Research avenue**
“Description”: Epidemiological research	Measuring the burden
	Understanding risk factors
	Evaluating the existing interventions
“Delivery”: Health policy and systems research	Studying capacity to reduce exposure to proven health risks
	Studying capacity to deliver efficacious interventions
“Development”: Improving existing interventions	Research to improve deliverability
	Research to improve affordability
	Research to improve sustainability
“Discovery”: Developing novel interventions	Basic research
	Clinical research
	Public health research

* Source: Rudan et al. [15].

### Database development

Once the search was completed, we used the citation report function on WoS to calculate h–indices for each time period and each geographic region and country. To compute h–index as described in our methods above, it was necessary to download all citation data into a Microsoft Excel format and extract the citation data for each individual paper during the chosen time period, while adding the three–year citation window. The sum of the number of citations per year would then be calculated for each publication.

These totals would then be ranked from highest to lowest and numbered accordingly. This allows the h–index to be calculated by reviewing where the highest rank number is greater than or equal to the corresponding number of citations. This process was repeated for each country for each of the three time periods and the results collated.

As WoS only allows data for 500 papers to be downloaded at once, this was a very time–consuming process. For countries producing more than 500 papers in the area of public health during the 5–year period, the citation information needed to be downloaded 500 papers at a time and then collated into a single data set. Furthermore, it was not possible to download the citation data for searches that produce more than 10 000 results. In all such cases, searches were split into years, and the results were further subdivided using marked lists to enable the citation data to be accessed.

### Data analysis

Once we collected the relevant citation data from WoS, we recorded the h–index, total number of publications and total number of citations for each country in a separate database. To perform all the planned analyses, the Gross Domestic Product (GDP) for each country was also recorded, using the World Bank's national–level estimates for the year 2010, or as close as possible, and recorded in US$ [16]. If this data was not available from the World Bank, alternative sources with best estimates were used, typically national estimates generated by the countries themselves and reported at the websites of their national governments.

We used the databases described above to rank all countries by their absolute number of publications and h–indices in each time period, to calculate and rank the absolute rate of increase in h–index between the first and last time period (which was only computed for those countries with an h–index in the first time period of ≥10), to rank all countries by their absolute number of publications per GDP for the most recent time period (for those countries whose number of publications ≥30), and by their h–index per GDP for the most recent time period (for those with an h–index of ≥10).

All papers that formed the h–core had the author’s address information reviewed and manually recorded for all contributing authors. The papers with multiple contributing authors were counted more than once when the co–authorship was cross–regional and inter–institutional. This was done through manual data extraction. The institutions to which the authors of h–core papers were affiliated to were recorded in a separate Microsoft Excel data set. Institutions were only verified as universities after a Google search was performed to investigate the institution type. The Universities that contributed more than 2 papers to the h–core were considered as making a notable contribution to global public health in their specific research environment.

## RESULTS

The results shall initially focus on describing the characteristics of public health research on a global scale, before focusing on the impact, measured using h–indices, within the 8 geographic regions, individual countries and at specific universities. This will be followed by the analysis of the distribution of papers in h–core by research topics and types of research used.

### Global level

**Figure 1:**
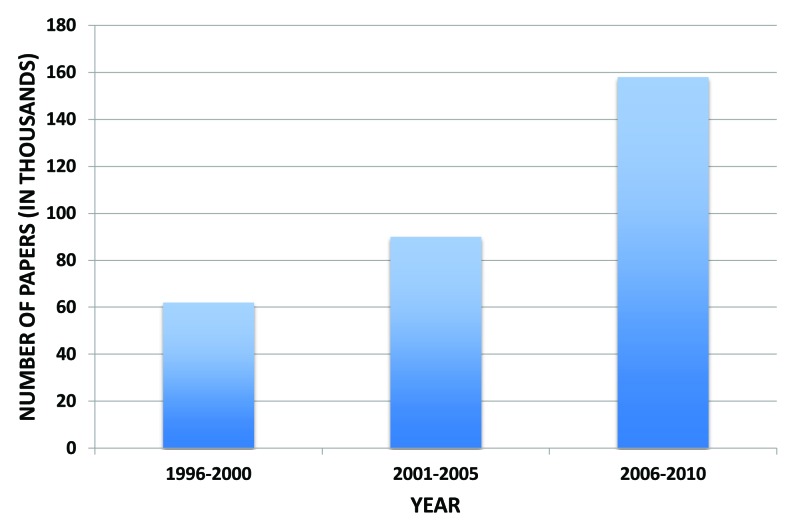
Total number of public health–related publications worldwide over 3 time periods.

The total number of papers that could be considered public health research has dramatically increased over the three time periods, from 63 571 (in 1996–2000) to 89 992 (in 2001–2005) and 158 938 (in 2006–2010). This is a 2.5–fold increase (**Figure 1**). Note that these values will slightly differ from the sum of each country’s publications because some papers were allocated to more than one country based on authors' affiliations.

### Regional level

**Figure 2:**
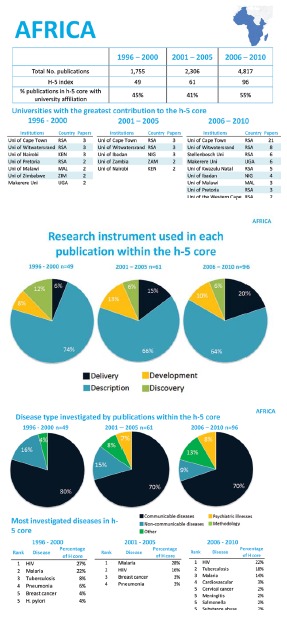
An assessment of capacity to conduct public health research for African region.

**Figure 3:**
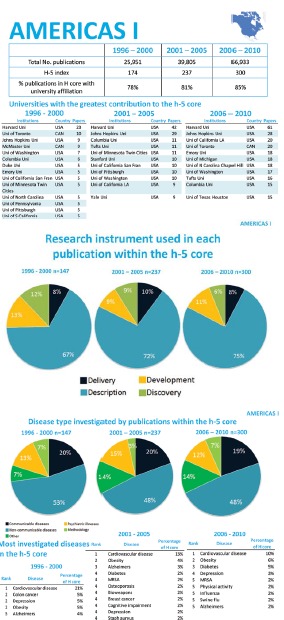
An assessment of capacity to conduct public health research for North–American region.

**Figure 4:**
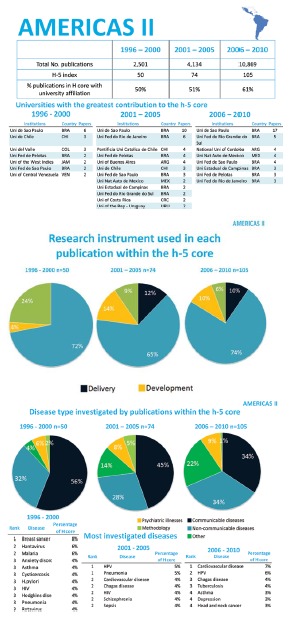
An assessment of capacity to conduct public health research for Latin–American region.

**Figure 5:**
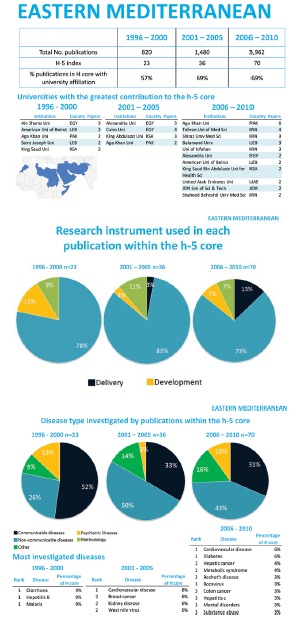
An assessment of capacity to conduct public health research for Eastern Mediterranean region.

**Figure 6:**
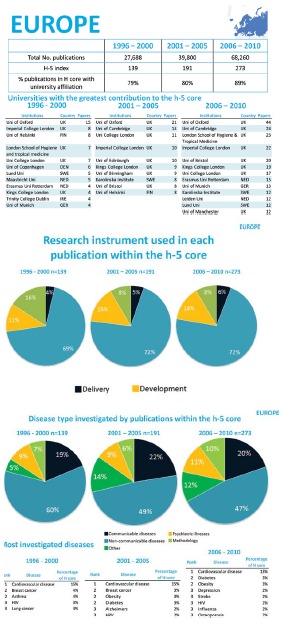
An assessment of capacity to conduct public health research for European region.

**Figure 7:**
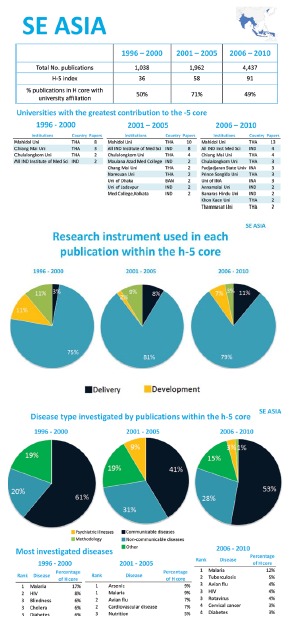
An assessment of capacity to conduct public health research for South–East Asian region.

**Figure 8:**
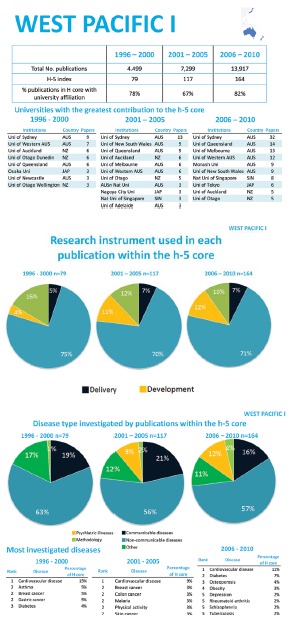
An assessment of capacity to conduct public health research for West Pacific I region.

**Figure 9:**
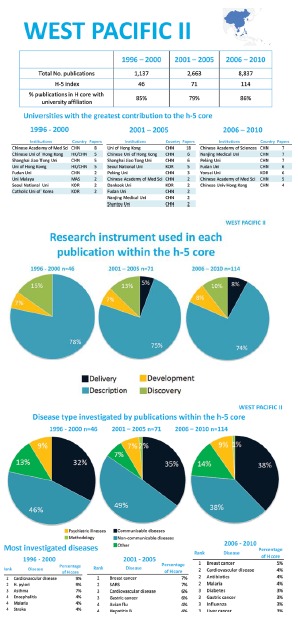
An assessment of capacity to conduct public health research for West Pacific II region.

As the eight regions differ with regard to their productivity in public health research and impact of their research, they shall be considered separately through an in–depth analysis to identify the hubs of research within those regions, as well as the topics of interest. **Figures 2 to 9** provide summary results in the form of a “fact sheet” for each region.

In the first time–period (1996–2000), the most productive region was Europe with 27 688 publications, closely followed by Americas I with 25 951 publications. This pattern is followed in the further two time periods, with Europe and Americas producing 68 260 and 66 933 publications in 2006–2010, respectively. The least productive region in 1996–2000 is the Eastern Mediterranean with 820 publications and the region remain lowest–ranked in 2006–2010 with 3962 publications. However, these regions do not have similar population sizes or number of countries. Therefore, the absolute rate of increase should also be considered in cross–regional comparisons. The region with the largest absolute increase in productivity is West Pacific II. The number of publications in that region increased from 1137 in 1996–2000 to 8837 in 2006–2010, representing an absolute increase of 677%. Europe had the lowest increase in publications during the same period, of 146% (**Table 2**).

**Table 2:** Absolute increase in number of publications and h–indices for each region

Region	Absolute increase in number of publications between 1996–2000 and 2006–2010 (%)	Absolute increase in h–index between 1996–2000 and 2006–2010 (%)
Africa	174	95
Americas I	158	72
Americas II	334	110
East Med	383	204
Europe	146	96
South–East Asia	327	152
West Pacific I	209	108
West Pacific II	677	148

The region with the highest h–index throughout all three time periods was Americas I. Their h–index increased from 174 to 300. However, they had the lowest absolute rate of increase in h–index, of 72% (**Table 2**). The Eastern Mediterranean Region (EMR) had the lowest h–index in all three periods – 23 (in 1996–2000), 36 (in 2001–2005) and 70 (in 2006–2010). However, they were also the region with the greatest increase in h–index, by 204%. In every region, the absolute increase in number of publications (productivity) was greater than the increase in h–index (**Table 2**).

### National level

**Figure 10:**
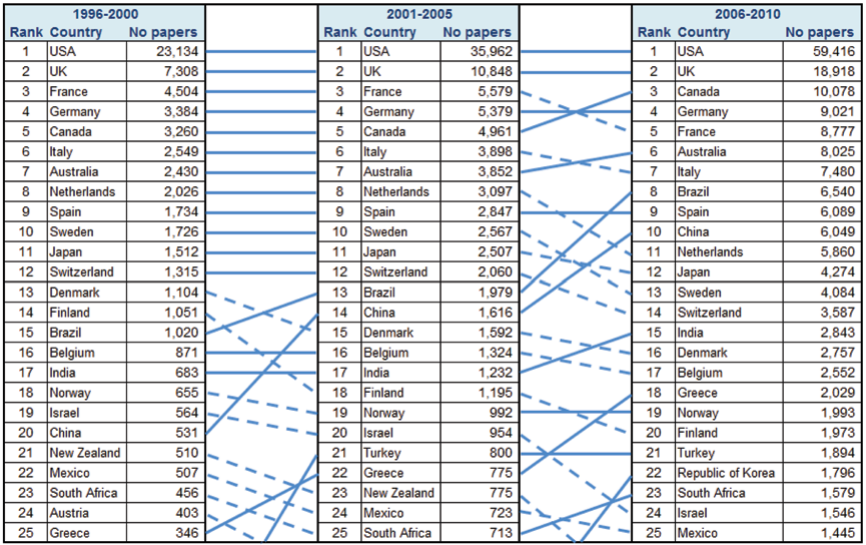
Top 25 countries ranked by total number of publications in each time period. Continuous blue lines indicate improvement in rank between the two periods or no change in rank. Dashed blue lines indicate decrease in rank between the two periods.

The countries were ranked by total number of publications over the three investigated five–year periods. **Figure 10** ranks the top 25 most productive countries over the three time periods. The complete set of results can be seen in **Online Supplementary Document(Online Supplementary Document)**. The USA dominates by a wide margin, with the UK, Canada, Germany and France consistently ranking in the top five. Of note is the overall improvement in productivity and well as ranking of some the BRICS nations, specifically Brazil and China, with South Africa making an entrance into the top 25 in 2006–2010.

**Figure 11:**
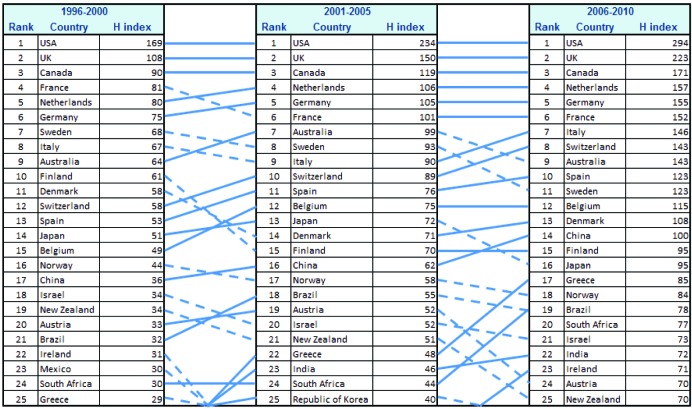
Top 25 countries ranked by h–index in each time period. Continuous blue lines indicate improvement in rank between the two periods or no change in rank. Dashed blue lines indicate decrease in rank between the two periods.

Considering the h–index of individual countries, it can be noted that the overall trend is an increase in h–index over the three time periods. **Figure 11** ranks the top 25 countries with the highest h–indices over the three time periods. The complete set of results can be seen in **Online Supplementary Document(Online Supplementary Document)**. The USA is the leading country on this list, but not to such a degree as in total publication number. Smaller European nations, such as Sweden, Finland and Switzerland, have risen up the ranks based on their h–index although they did not feature as highly in total publication number. The BRICS nations continue to improve, particularly China, Brazil and India, with both an increase in quantity of papers and h–index.

To explore which nations were the most successful throughout the entire study period in improving their capacity for research, the absolute rate of increase for nations with an original h–index greater than 10 between the 1995–2000 hours–index and the 2006–2010 hours–index was calculated. Estonia and Pakistan are at the top of the rankings, with an absolute rate of increase of 230%. In comparison, the USA’s rate of increase was 74% and the UK’s 106%. The only countries found to have a negative rate of change between the two time periods were Jamaica (with a decrease of 15%) and Guinea–Bissau (with a decline of 20%).

The total number of publications in relation to GDP was considered for the 2006–2010 period. To avoid spurious results, only countries with more than 30 publications were included. The 25 countries that were most productive in relation to their GDP are ranked in **Table 3**, and the full results can be found in **Online Supplementary Document(Online Supplementary Document)**. African Nations dominate the top 25 ranks, indicating that some of them are being very productive with limited resources, particularly the Gambia – whose GDP is amongst the lowest worldwide.

**Table 3:** Top 25 countries ranked by total number of publications/gross domestic product (GDP) in 2006–2010

Rank	Country	GDP (2010, in US$ billion)	Papers in 2006–2010	Papers per GDP
1	Gambia	0.78	73	93.1
2	Malawi	3.29	143	43.5
3	Uganda	13.36	326	24.4
4	Zimbabwe	5.20	122	23.5
5	Kenya	23.53	482	20.5
6	Burkina Faso	7.11	127	17.9
7	Tanzania	19.72	341	17.3
8	Iceland	16.39	259	15.8
9	Senegal	10.37	159	15.3
10	Nepal	10.10	148	14.6
11	Mongolia	3.45	47	13.6
12	Zambia	9.80	131	13.4
13	Ghana	14.80	197	13.3
14	Estonia	13.90	181	13.0
15	Lao	4.02	52	12.9
16	Benin	5.23	61	11.7
17	Ethiopia	20.40	235	11.5
18	Cambodia	8.69	100	11.5
19	New Zealand	120.04	1361	11.3
20	Croatia	45.87	506	11.0
21	Cameroon	19.21	210	10.9
22	Rwanda	3.79	41	10.8
23	Denmark	256.82	2757	10.7
24	Mozambique	9.13	96	10.5
25	Madagascar	5.76	60	10.4

The h–index was then reviewed in relation to GDP and the list of top 25 countries is shown in **Table 4**. Full results are available in **Online Supplementary Document(Online Supplementary Document)**. As with the absolute rate of increase, only countries with an h–index greater than 10 in 2006–2010 were considered. The upper ranks are again dominated by African nations, whilst the USA now ranks second to last. The Gambia has again performed particularly well, indicating that they are producing high quality research with limited resources. This is clearly a result of the research activity of a well–known international research centre, supported largely by the Medical Research Council in the UK, that was established in the Gambia in the 20th century.

**Table 4:** Top 25 countries ranked by h–index/ gross domestic product (GDP) in 2006–2010

Rank	Country	GDP (2010, in US$ billion)	H–index for 2006–2010	H–index per GDP
1	Gambia	0.78	22	28.1
2	Malawi	3.29	29	8.8
3	Lao	4.02	18	4.5
4	Zimbabwe	5.20	23	4.4
5	Rwanda	3.79	15	4.0
6	Fiji	3.03	12	4.0
7	Niger	4.38	17	3.9
8	Burkina Faso	7.11	25	3.5
9	Mali	6.97	23	3.3
10	Iceland	16.39	54	3.3
11	Mongolia	3.45	11	3.2
12	Madagascar	5.76	18	3.1
13	Uganda	13.36	39	2.9
14	Benin	5.23	14	2.7
15	Cambodia	8.69	23	2.6
16	Papua New Guinea	6.55	17	2.6
17	Malta	6.65	17	2.6
18	Barbados	4.03	10	2.5
19	Mozambique	9.13	22	2.4
20	Estonia	13.90	33	2.4
21	Senegal	10.37	24	2.3
22	Nepal	10.10	23	2.3
23	Zambia	9.80	22	2.2
24	Kenya	23.53	52	2.2
25	Gabon	9.68	19	2.0

In the period 2006–2010, it was noted that there was a considerable gap between the country at the top of the rankings and all others from the same region in the number of publications and h–index. In the African region, South Africa was at the top (1579 publications and h–index of 77); in Americas I, the USA (59 416 publications and h–index of 294); in Americas II, Brazil (6540 publications and h–index of 78); in East Mediterranean, Iran (1326 publications and h–index of 42); in Europe, the UK (publications 18 918, h–index 223); in SE. Asia, India (2843 publications; h–index of 72); in West Pacific I, Australia (8025 publications; h–index 143); and in West Pacific II, China (6049 publications; h–index 100). When h–index is considered in relation to GDP, the only country that remains at the top within its own region is India – which has the highest h–index per GDP in SE. Asia. The remaining countries all moved down their regional rankings, because other nations with lower total publications and h–indices perform better in relation to their GDP. Countries which are particularly successful in relation to their GDP are the Gambia, Malawi, Barbados, Nicaragua, Jordan, Lebanon, Iceland, Estonia, Thailand, Laos and Mongolia.

### Sub–national level

In general, the percentage of papers in regional h–cores that were originated at a regional university increased throughout the three time periods. The exceptions were SE. Asia and West Pacific II, where the percentages in the first and the last time period were very similar. The region with the greatest university contribution to the regional h–core was Europe, where 89% of h–core publications had authorship from a European university. This was similar in other regions with high–income countries, such as Americas I (with 85%) and West Pacific I (with 82%). However, in poorer regions, the percentage of papers in the h–core originated from a regional university was lower. In SE. Asia, only 49% of papers were university–based in 2006–2010, and in Africa they contributed to 55%. Some of the leading regional universities are Cape Town's, Harvard, Universidade de Sao Paulo, Oxford, Madihol and Sydney's.

### Types of research

The four instruments (or “domains”) of health research, as described by Rudan [15], could be summarized as “the four D's”: “description”, “delivery”, “development” and “discovery”. We categorized each paper that contributed to the regional h–core in each time period into one of those four domains. The results for each individual region can be seen in **Figures 2 to 9**. In each region, the majority of papers in the h–core were “descriptive” papers – ranging from 64% (in West Pacific I) to 79% (in South–East Asia). In all regions, the proportion of research in the h–core relating to “discovery” research decreased, with the exception of Eastern Mediterranean region (EMR) where it remained stable. There was little change in the proportion of research that related to “development”, but in the majority of regions, research on “delivery” in public health increased (the only exceptions being Americas I and West Pacific I).

### Topics of research

Each publication that related to a disease in a region’s h–core throughout the three time periods was classified into non–communicable diseases (NCDs), infectious diseases (ID), other diseases, or a predominantly methodological papers. In three regions, NCDs were the topic of most interest in the h–core throughout all three time periods: Americas I, Europe and West Pacific I. In two regions, the research interest was mainly focused on infectious diseases throughout all three periods: Africa and South–East Asia. In the remaining three regions (Americas II, Eastern Mediterranean and West Pacific II), a similar pattern can be seen – the proportion of papers relating to communicable diseases is decreasing, and the proportion relating to NCDs is increasing (**Figures 2 to 9**).

The specific diseases under investigation by the publications in the h–core follow a similar pattern to the proportion of topics. Cardiovascular diseases were most frequently studied in high–income regions, and increasing in importance in regions with lower income. Moreover, in high–income regions, diabetes, obesity and depression are increasing in importance. Overall, there is a slight increase in the proportion of papers relating to psychiatric illnesses, with the greatest increase in the West Pacific I. In Europe, papers relating to the methodology of performing public health research are increasing.

## DISCUSSION

Increasing investment in global public health research has resulted in a need to understand where capacity to perform research lies. Currently, some areas of the world may not be seen as “worthy” of research investment by some funders. However, there is a lack of an established and effective methodology that can be used to identify the nations and institutions that are demonstrating an improved capacity for public health research globally. This study was successful in developing a new bibliometric approach to address this question, by adapting the h–index to allow research capacity in public health worldwide to be assessed over time. The results clearly highlight countries that improved their capacity for public health research and the institutions that are contributing substantially to public health research. In addition, this study has been successful in providing an understanding of the trends in research instruments (“domains”) used and topics that were investigated through public health research.

This study has, therefore, not only established a methodology to assess public health research capacity worldwide, but also provided a baseline to which future evaluations can be compared. In addition, the methodology developed here could be adapted to any other topic of scientific research in order to assess global, regional, national and sub–national capacity for research.

On viewing the total number of publications and h–indices over the three time periods for each region, it can be seen that the large majority of papers come from European and America I regions. However, despite the large numbers of publications, their absolute increase over time in both number of publications and h–index is relatively low. Other regions, such as Eastern Mediterranean and South–East Asia, are showing a considerable improvement in both publication number and h–index. At the same time, Western Pacific II region has seen a huge absolute increase in publications, but the increase in h–index is not correspondingly high. Africa has a fairly low absolute increase in both publication number and h–index, with low values to start from, too.

The USA clearly dominates in terms of productivity and h–index. However, when GDP is taken into account, the USA actually ranks rather low. In comparison to the UK, which consistently ranks second in terms of both quantity and quality, the USA is producing a huge amount of research, yet their h–index is not correspondingly high. At the same time, the BRICS nations have been making substantial improvements, all of them ranked in the top 25 countries for productivity and h–index in 2006–2010 period, with the exception of Russia. They all had absolute rates of increase in h–index greater than 140% except Russia, whose rate of increase was only 44%. It is possible this could be explained by the frequency at which countries publish in the English language. As reported in the literature review, non–English language journals are less frequently indexed in WoS. Some nations may appear not to be performing well, when in fact it is simply that their research is predominantly published in non–English language journals. This has been reported to be the case for Russia in stroke–related research [17]. However, this could also be the case for many other countries, whose research capacity is being under–represented in this analysis. Furthermore, as non–English language papers are less likely to be cited [18], they may incorrectly appear to be of lower quality whenever citations are used as a partial indicator of research quality.

Despite low numbers of publications and low h–indices in general, African nations can be seen to be performing well, considering the resources available (measured by GDP). However, similar to South–East Asia, about half of the papers in the h–core for the region have been produced by non–university institutions. It is, therefore, likely that international research organisations are performing large portion of this regional research, which may inhibit the progress of local universities. For example, in Egypt, the US Navy performed much of the research in the h–core. However, in the majority of regions, the proportion of non–university authored publications in the h–core is declining, suggesting that university–based research is improving in quality almost universally.

On reviewing the research topics that occur in the h–core of the regions, it can be noted that Africa and South–East Asia are the only two regions where communicable diseases remain proportionally the most studied in the 2006–2010 time–period. The Eastern Mediterranean, America II and West Pacific II regions can be seen as transitioning from their historic focus on communicable diseases to NCDs, whilst Europe and America I have a very similar distribution of research throughout. Regarding research instruments (“domains”), both Rudan et al. and Leroy et al. proposed that too much research funding may be allocated to the development of new interventions, which could not be as effective in reducing child mortality as implementing the existing interventions effectively [15,19]. It is, therefore, pleasing to see an increase in research related to delivery of interventions, whilst research relating to novel discoveries is decreasing, thus achieving a more desirable balance. In Africa in particular, research on delivery of public health interventions is increasing in both quality and quantity, demonstrating the capacity in this region to improve implementation of available interventions.

The key strength of our study lies in the methodology developed, which allowed not only an assessment of global public health research capacity, but also the trends over time. This was the first application of this novel methodology, using existing large data sets on WoS in a novel way, allowing the emerging research hubs to be identified and the current research trends to be visualised. The use of the three–year citation window following each 5–year period ensured that studies towards the end of the time–period had adequate time to be cited. Furthermore, in the validation of 2654 articles that contributed to the regional h–indices throughout the 3 time–periods, 2% were found to not be relevant to public health. This was felt to be an acceptable level of specificity. When considering the possible biases related to sensitivity of the proposed approach to literature search, whilst there are undoubtedly public health papers that remained unidentified using our search strategy, we find it unlikely that this problem could affect the overall results or rankings of nations that we reported here, and which seem plausible to a large extent.

The novel use of the h–index proposed in this study has provided a single measure with which the quality and quantity of research produced by regions, nations and institutions can be compared over time. Whilst the h–index is superior to citations per paper and IF, it does have its limitations. As an example, it does not provide an understanding of the proportion of low quality studies produced by a country or region. In the case of the USA, this could be particularly interesting, as their h–index is very high, yet they have a vast number of publications which do not contribute to it, which is proportionally much greater than other nations. There is also a possible concern about the phenomenon known as the “Matthew effect”, where more recognised and established researchers may have their work cited more, simply due to name recognition rather than the true quality of the publication [20]. This would falsely inflate the apparent gap between more established research nations and those that are emerging. In addition, it has been shown that the h–index is higher when there is more international collaboration between nations [21]. As a metric, it therefore disadvantages those LMIC who do not have as much opportunity for collaboration as North America and Europe. This would, again, act to increase apparent inequalities between established and emerging research nations.

As with many bibliometric–type studies, this study has limitations that are inherent in using an online database to access citation data. These databases have language bias, with papers and journals not writing in English less likely to be indexed. The result would be fewer publications from emerging research countries, where research is more likely not to be published in English. Another problem was studied by Gingras, who noted that some wealthy institutions from middle–income countries may be able to manipulate their citation numbers by offering highly cited researchers attractive contracts for minimal work if they would agree to affiliate themselves with the paying university as the secondary affiliation. Gingras describes these as “dummy affiliations”, with no real impact on teaching and research in universities, allow marginal institutions to boost their position in the rankings of universities without having to develop any real scientific activities [22].

There are also many academics who view the use of citation metrics to measure quality of research as “a terrible idea”. Sabaratnam and Kirby wrote a response to the Higher Education Funding Council for England, who were considering using citation metrics when assessing research quality, and received over 200 signatories objecting to the idea [23]. They quite rightly pointed out that a citation is not necessarily an endorsement of quality. They state that all methods currently available to assess quality are flawed. Whilst the h–index is certainly not a perfect measure of research quality or capacity, it seems that it may be the best currently available. The fact that there is not a perfect measurement technique does not mean that no attempt should be made to understand public health research capacity, and identify those who are improving.

Hirsch himself believed that “a single number can never give more than a rough approximation to an individual’s multifaceted profile, and many other factors should be considered in combination in evaluating an individual” [24]. It is certainly not possible that a single metric, such as h–index, can truly describe an institution or country’s contribution to global public health research. However, this study provides a bibliometric profile of regions, countries and institutions which, when viewed together, can characterise their publication and research efforts and provide an indication of their capacity to perform public health research. Despite the limitations of bibliometric research, this study has been successful in identifying nations in each region which have capacity for public health research, which are improving and which are performing well despite limited resources.

Many of the nations seem to be improving both the quality and the quantity of their public health research with comparatively limited resources. Whilst some of these countries were expected to be making improvements, based on their rapid economic development (such as Brazil, South Africa, China and India), there have also been other unexpected nations demonstrating great capacity for public health research. Some, like Estonia and Pakistan, have made huge strides in improving their research quality and quantity. Others, like the Gambia, Malawi and Laos are producing high quality research despite extremely limited domestic resources. In addition, those universities which are contributing substantially to national research capacity should be recognised and supported.

We mentioned in the introduction section that the use of a country’s GDP for expenditure on health research is a proxy, as there is no other reliable method to track such expenditures. In light of this knowledge, social and political differences (such as war, conflict, or financial instability) between countries or regions might also make it a challenge in figuring out how governments spend money on health research [25].

In the future, public health research shall likely become increasingly specialized, which may result in cutting–edge research becoming more expensive and based on large–scale “biobanks”. Therefore, identifying universities that perform well in all regions and increasing international communication and cooperation will be beneficial to the global public health research community. In many of the low–income countries, there is also a discrepancy between their current disease burden and the ability to perform public health research. Their universities should further focus on studying delivery of the existing public health interventions, to allow evidence–based decisions to be made based on locally relevant research. Increasing collaboration between LMICs and forming so–called “South–South partnerships” to address common health problems would also be beneficial, with a focus on those diseases that contribute significantly to national disease burdens, such as diabetes and cardiovascular disease. Ranasinghe argued that researchers in LMIC face additional challenges when attempting to publish their research, which is largely due to language and funding issues [26]. Therefore, medical journals should be encouraged to provide researchers throughout the world with equal opportunity to publish their research, and offer guidance how to improve its quality.

In the future, this study should be repeated at five–yearly intervals to identify new and emerging hubs of public health research. In order for future studies to be completed more efficiently, there are a number of steps that Web of Science^TM^ (WoS) itself could take to make the process more streamlined. It would be very beneficial to allow citation data to be collected for those searches which have >10 000 results. As the quality of research continues to grow, there will soon be many countries who produce >10 000 public health publications in a 5–year period. In addition, removing the cap, which only allows the citation data of 500 publications to be downloaded at a time, would be helpful. As some countries have over 50 000 publications to be analysed, collating all these results is extremely time consuming and could easily be avoiding by some simple adjustments by WoS. This methodology could also be extended to other fields of science, allowing them to assess the development of research capacity worldwide. However, it should be remembered that the evaluations of different fields based on h–indices are often not comparable, primarily due to large differences in the number of participating researchers and an overall number of citations.

## CONCLUSION

This is an exciting time for public health research. The potential funding available for research is larger than ever, allowing the quantity of research to increase, and the quality to improve. However, there is a danger that funding will continue to be allocated mainly to established and traditional “hubs” of research. In recent years, many nations, particularly LMIC, have been improving their research quantity and quality – thereby gaining capacity for public health research. This study was successful in developing a methodology, based on the h–index, which provides an assessment of capacity for public health research from 1996–2010. As expected, the USA and UK dominated public health research globally. However, there were a number of countries with limited resources, demonstrating improved capacity for public health research. In addition, university contributions to high quality research were increasing. There has been a shift in research domains – with more research on improving deliverability of existing interventions. The research being performed is also more representative of the burden of disease worldwide, with a shift towards NCDs. In order to improve the overall quality of public health research, international collaborations should be encouraged, while medical journals should seek to ensure that publication is a fair and equitable process.
