# Supplementary material for: Assessing global, regional, national and sub–national capacity for public health research: a bibliometric analysis of the Web of ScienceTM in 1996–2010
Source: J Glob Health. 2016 Jun 20;6(1):010504. doi: 10.7189/jogh.06.010504 (PMC4920005; doi:10.7189/jogh.06.010504)
Supplement: Online Supplementary Document [file jogh-06-010504-s001.pdf]

Appendix 1
The 8 regions used in the study and the countries included within each one

Appendix 2
Full results – total number of publications over the three five year periods by country

Appendix 3
Full results – h-index over the three five year periods

Appendix 4
Full results – total number of publications/GDP in 2006-2010

Appendix 5
Full results – h-index/GDP in 2006-2010

Appendix 1

The 8 regions used in the study and the countries included within each one

Africa

Algeria	Ethiopia	Niger
Angola	Gabon	Nigeria
Benin	Gambia	Rwanda
Botswana	Ghana	Sao Tome and Principe
Burkina Faso	Guinea	Senegal
Burundi	Guinea Bisseau	Seychelles
Cameroon	Kenya	Sierra Leone
Cape Verde	Lesotho	South Afriac
Central African Republic	Liberia	Swaziland
Chad	Madagascar	Togo
Comoros	Malawi	Uganda
Cote D'Ivoire	Mali	Tanzania
Congo	Mauritius	Zaire
Equatorial Guinea	Mozambique	Zambia
Eritrea	Namibia	Zimbabwe

W Pacific II

Cambodia	Malaysia	Phillipines
China	Marshall Islands	South Korea
Hong Kong	Micronesia	Samoa
Cook Islands	Mongolia	Solomon Islands
Fiji	Nauru	Tonga
Kiribati	Nieu	Tuvalu
Laos	Palau	Vanuatu
	Papua New Guinea	Vietnam

America I

Canada	Cuba	USA

W Pacific I

Australia	Japan	Singapore
Brunei	New Zealand	

East Mediterranean

Afghanistan	Kuwait	Saudi Arabia
Bahrain	Lenanon	Somalia
Djibouti	Libya	Sudan
Egypt	Morocco	Syria
Iran	Oman	Tunisia
Iraq	Pakistan	United Arab Emirates
Jordan	Qutar	Yemen

Europe

Albania	Greenland	Netherlands
Andorra Armenia	Hungary	Norway
Austria	Iceland	Poland
Azerbaijan	Ireland	Portugal
Belarus	Israel	Romainia
Belgium	Italy	Russia
Bosnia	Kazakhstan	San Marino
Bulgaria	Kosavo	Serbia
Croatia	Kyrgyzstan	Slovakia
Cyprus	Latvia	Spain
Czech Republic	Liechtenstein	Sweden
Denmark	Lithuania	Switzerland
Estonia	Luxembourg	Tajikistan
Finland	Macedonia	Turkey
France	Malta	Turkmenistan
Georgia	Moldova	Ukraine
Germany	Monaco	United Kingdom
Greece	Montenegro	Uzbekistan
		Yugoslavia

SE Asia

Bangladesh	Maldives	North Korea
Bhutan	Myanmar	Sri Lanka
India	Nepal	Thailand
		Timor-L'este

America II

Antigue and Barbuda	Dominica	Mexico
Argentina	Dominican Republic	Nicaragua
Bahamas	Ecuador	Panama
Barbados	El Salvador	Paraguay
Belize	Grenada	Peru
Bolivia	Guatemala	St Kitts and Nevis
Brazil	Guyana	St Lucia
Chile	Haiti	St Vincent + the Grenadines
Columbia	Honduras	Suriname
Costa Rica	Jamaica	Trinidad and Tobago
		Uruguay
		Venezuela

Appendix 2

Full results – total number of publications over the three five year periods

Country	Total number of publications		
	1996-2000	2001 - 2005	2006 - 2010
Afghanistan	1	6	23
Albania	5	22	41
Algeria	14	20	86
Andorra	0	0	1
Angola	3	5	20
Antigua and Barbuda	0	1	1
Argentina	310	468	961
Armenia	3	4	20
Australia	2430	3852	8025
Austria	403	688	1360
Azerbaijan	1	3	4
Bahamas	1	0	1
Bahrain	3	13	29
Bangladesh	70	130	302
Barbados	18	21	31
Belarus	6	2	2
Belgium	871	1324	2552
Belize	2	2	5
Benin	15	27	61
Bhutan	0	0	2
Bolivia (Plurinational State of)	34	32	70
Bosnia and Herzegovina	4	12	121
Botswana	9	15	47
Brazil	1020	1979	6540
Brunei Darussalam	1	1	7
Bulgaria	56	64	173
Burkina Faso	56	75	127
Burundi	9	2	11
Cambodia	5	32	100
Cameroon	81	131	210
Canada	3260	4961	10078
Cape Verde	0	0	4
Central African Republic	13	10	22
Chad	5	9	11
Chile	252	338	619
China	531	1616	6049
Colombia	1	6	10
Comoros	0	0	5
Congo	20	27	57
Cook Islands	0	0	1
Costa Rica	42	95	137
Côte d'Ivoire	77	67	90

Country	Total number of publications 1996-2000	2001 - 2005	2006 - 2010
Croatia	101	265	506
Cuba	52	95	177
Cyprus	11	18	85
Czech Republic	148	256	581
Democratic People's Republic	0	0	1
Denmark	1104	1592	2757
Djibouti	1	2	1
Dominica	0	0	3
Dominican Republic	6	14	26
Ecuador	29	50	110
Egypt	128	197	459
El Salvador	3	8	18
Equatorial Guinea	2	2	5
Eritrea	3	5	7
Estonia	65	89	181
Ethiopia	85	120	235
Fiji	6	6	29
Finland	1051	1195	1973
France	4504	5579	8777
Gabon	19	32	64
Gambia	43	45	73
Georgia	8	12	41
Germany	3384	5379	9021
Ghana	61	90	197
Greece	346	775	2029
Grenada	1	6	11
Guatemala	30	33	72
Guinea	5	15	17
Guinea-Bissau	15	21	16
Guyana	2	3	12
Haiti	5	19	20
Honduras	12	21	27
Hungary	168	315	571
Iceland	97	123	259
India	683	1232	2843
Indonesia	56	103	209
Iran (Islamic Republic of)	44	205	1326
Iraq	6	12	37
Ireland	263	395	955
Israel	564	954	1546

	Total number of publications		
Country	1996-2000	2001 - 2005	2006 - 2010
Italy	2549	3898	7480
Jamaica	45	68	90
Japan	1512	2507	4274
Jordan	54	87	132
Kazakhstan	9	20	33
Kenya	213	251	482
Kiribati	0	0	0
Kuwait	56	94	154
Kyrgyzstan	0	6	22
Lao People's Democratic Repu	1	16	52
Latvia	10	25	49
Lebanon	119	73	183
Lesotho	1	3	7
Liberia	1	3	2
Libya	10	12	23
Lithuania	24	55	187
Luxembourg	21	37	115
Madagascar	39	27	60
Malawi	40	56	143
Malaysia	74	125	445
Maldives	0	0	2
Mali	17	40	72
Malta	24	12	47
Marshall Islands	0	1	1
Mauritania	7	6	5
Mauritius	4	8	12
Mexico	507	723	1445
Micronesia (Federated States	2	1	4
Monaco	1	3	10
Mongolia	6	20	47
Montenegro	-	-	15
Morocco	76	106	198
Mozambique	11	17	96
Myanmar	3	13	20
Namibia	9	4	9
Nauru	0	0	0
Nepal	35	66	148
Netherlands	2026	3097	5860
New Zealand	510	775	1361
Nicaragua	20	18	45

Country	Total number of publications		
	1996-2000	2001 - 2005	2006 - 2010
Niger	19	17	43
Nigeria	139	174	616
Niue	0	0	1
Norway	655	992	1993
Oman	19	34	53
Pakistan	58	123	462
Palau	1	0	2
Panama	6	11	36
Papua New Guinea	34	37	50
Paraguay	7	14	32
Peru	67	131	335
Philippines	73	107	235
Poland	258	522	1215
Portugal	154	299	898
Qatar	3	22	75
Republic of Korea	229	656	1796
Republic of Moldova	6	6	15
Romania	48	104	415
Russian Federation	310	392	656
Rwanda	2	2	41
Saint Kitts and Nevis	0	0	6
Saint Lucia	2	0	2
Saint Vincent and the Grenadi	0	0	1
Samoa (western)	1	0	2
San Marino	1	0	0
Sao Tome and Principe	2	1	2
Saudi Arabia	203	258	338
Senegal	99	124	159
Serbia	-	-	284
Seychelles	9	4	13
Sierra Leone	4	3	15
Singapore	125	381	827
Slovakia	68	166	272
Slovenia	64	133	287
Solomon Islands	1	4	7
Somalia	0	0	1
South Africa	456	713	1579
South Sudan	33	55	92
Spain	1734	2847	6089
Sri Lanka	17	48	146

Country	Total number of publications		
	1996-2000	2001 - 2005	2006 - 2010
Sudan	33	55	92
Suriname	0	3	5
Swaziland	0	4	5
Sweden	1726	2567	4084
Switzerland	1315	2060	3587
Syrian Arab Republic	5	20	38
Tajikistan	0	2	9
Thailand	203	433	958
The former Yugoslav Republic	5	8	38
Timor-Leste	0	0	0
Togo	20	16	27
Tonga	0	0	8
Trinidad and Tobago	1	48	74
Tunisia	63	161	376
Turkey	198	800	1894
Turkmenistan	0	1	1
Tuvalu	0	0	0
Uganda	81	117	326
Ukraine	33	46	77
United Arab Emirates	29	55	158
United Kingdom	7308	10848	18918
United Republic of Tanzania	101	159	341
United States of America	23134	35962	59416
Uruguay	51	67	126
Uzbekistan	2	12	30
Vanuatu	2	2	3
Venezuela (Bolivarian Republi	128	144	366
Viet Nam	51	145	316
Yemen	11	14	27
Zambia	33	46	131
Zimbabwe	97	84	122

Appendix 3

Full results – h-index over the three five year periods

Country	1996-2000 H index	2001-2005 H index	2006-2010 H index
Afghanistan	1	5	11
Albania	2	8	9
Algeria	5	6	17
Andorra	0	0	1
Angola	3	4	10
Antigua and Barbuda	0	1	1
Argentina	24	34	55
Armenia	2	1	7
Australia	64	99	143
Austria	33	52	70
Azerbaijan	0	0	2
Bahamas	1	0	0
Bahrain	3	5	9
Bangladesh	17	24	34
Barbados	9	8	10
Belarus	3	2	2
Belgium	49	75	115
Belize	1	0	3
Benin	5	11	14
Bhutan	0	0	2
Bolivia (Plurinational	12	11	20
Bosnia and Herzegovina	3	4	9
Botswana	2	7	14
Brazil	32	55	78
Brunei Darussalam	1	1	3
Bulgaria	12	14	26
Burkina Faso	14	15	25
Burundi	4	2	9
Cambodia	4	8	23
Cameroon	15	21	30
Canada	90	119	171
Cape Verde	0	0	2
Central African Republic	6	4	9
Chad	2	6	7
Chile	21	31	39
China	36	62	100
Colombia	1	3	7
Comoros	0	0	4
Congo	6	8	17
Cook Islands	0	0	1
Costa Rica	12	21	25
Côte d'Ivoire	13	16	25
Croatia	8	19	37

Country	1996-2000 H index	2001-2005 H index	2006-2010 H index
Cuba	8	13	24
Cyprus	4	6	17
Czech Republic	18	33	50
Democratic People's	0	0	1
Denmark	58	71	108
Djibouti	1	2	0
Dominica	0	0	3
Dominican Republic	4	7	11
Ecuador	11	14	22
Egypt	13	21	34
El Salvador	1	3	5
Equatorial Guinea	2	2	4
Eritrea	2	4	5
Estonia	10	19	33
Ethiopia	10	15	25
Fiji	3	3	12
Finland	61	70	95
France	81	101	152
Gabon	8	11	19
Gambia	16	16	22
Georgia	3	5	11
Germany	75	105	155
Ghana	12	18	27
Greece	29	48	85
Grenada	1	4	6
Guatemala	15	10	18
Guinea	5	7	9
Guinea-Bissau	10	11	8
Guyana	1	2	7
Haiti	3	8	8
Honduras	8	7	10
Hungary	17	32	53
Iceland	19	28	54
India	25	46	72
Indonesia	13	19	33
Iran (Islamic Republic of)	8	19	42
Iraq	3	4	9
Ireland	31	37	71
Israel	34	52	73
Italy	67	90	146
Jamaica	13	11	11
Japan	51	72	95
Jordan	8	10	17

Country	1996-2000 H index	2001-2005 H index	2006-2010 H index
Kazakhstan	4	9	11
Kenya	24	31	52
Kiribati	0	0	0
Kuwait	11	12	21
Kyrgyzstan	0	2	9
Lao People's Democratic	0	8	18
Latvia	3	11	18
Lebanon	26	13	29
Lesotho	1	3	2
Liberia	1	2	1
Libya	3	5	8
Lithuania	7	12	22
Luxembourg	10	14	27
Madagascar	8	9	18
Malawi	13	16	29
Malaysia	15	22	37
Maldives	0	0	2
Mali	7	11	23
Malta	8	9	17
Marshall Islands	0	0	1
Mauritania	3	3	3
Mauritius	2	3	5
Mexico	30	37	59
Micronesia (Federated	1	1	2
Monaco	0	2	9
Mongolia	5	9	11
Montenegro	-	-	5
Morocco	6	11	18
Mozambique	4	10	22
Myanmar	2	5	9
Namibia	4	3	4
Nauru	0	0	0
Nepal	11	16	23
Netherlands	80	106	157
New Zealand	34	51	70
Nicaragua	9	7	14
Niger	6	6	17
Nigeria	13	18	31
Niue	0	0	1
Norway	44	58	84
Oman	5	9	12
Pakistan	10	18	33
Palau	1	0	1

Country	1996-2000 H index	2001-2005 H index	2006-2010 H index
Panama	1	6	14
Papua New Guinea	13	13	17
Paraguay	4	6	15
Peru	14	22	35
Philippines	12	18	27
Poland	25	36	63
Portugal	21	31	56
Qatar	2	5	16
Republic of Korea	25	40	64
Republic of Moldova	3	4	5
Romania	10	17	31
Russian Federation	25	31	36
Rwanda	2	1	15
Saint Kitts and Nevis	0	0	6
Saint Lucia	1	0	2
Saint Vincent and the	0	0	1
Samoa (western)	1	0	2
San Marino	1	0	0
Sao Tome and Principe	2	1	1
Saudi Arabia	12	19	26
Senegal	14	20	24
Serbia	-	-	22
Seychelles	5	3	8
Sierra Leone	2	2	7
Singapore	21	36	62
Slovakia	15	23	34
Slovenia	11	19	29
Solomon Islands	1	3	4
Somalia	0	0	1
South Africa	30	44	77
South Sudan	7	15	17
Spain	53	76	123
Sri Lanka	6	16	23
Sudan	7	15	17
Suriname	0	2	4
Swaziland	0	2	4
Sweden	68	93	123
Switzerland	58	89	143
Syrian Arab Republic	3	8	12
Tajikistan	0	1	6
Thailand	26	39	56
The former Yugoslav	2	4	8
Timor-Leste	0	0	0

Country	1996-2000 H index	2001-2005 H index	2006-2010 H index
Togo	5	5	5
Tonga	0	0	7
Trinidad and Tobago	1	12	11
Tunisia	9	14	24
Turkey	18	31	48
Turkmenistan	0	0	1
Tuvalu	0	0	0
Uganda	16	24	39
Ukraine	4	10	19
United Arab Emirates	6	11	21
United Kingdom	108	150	223
United Republic of	18	26	36
United States of America	169	234	294
Uruguay	17	21	23
Uzbekistan	2	6	8
Vanuatu	2	2	3
Venezuela (Bolivarian	12	18	31
Viet Nam	12	25	34
Yemen	3	4	10
Zambia	12	14	22
Zimbabwe	14	18	23

Appendix 4

Full results – total number of publications/GDP in 2006-2010

Country	2010 GDP USDbn	No papers	No papers / GDP
Albania	10.73	41	3.82
Algeria	116.51	86	0.74
Argentina	293.70	961	3.27
Australia	797.44	8025	10.06
Austria	325.55	1360	4.18
Bangladesh	81.47	302	3.71
Barbados	4.03	31	7.69
Belgium	400.38	2552	6.37
Benin	5.23	61	11.66
Bolivia	11.95	70	5.86
Bosnia and Herzegovina	12.80	121	9.45
Botswana	12.12	47	3.88
Brazil	1,096.75	6540	5.96
Bulgaria	32.99	173	5.24
Burkina Faso	7.11	127	17.87
Cambodia	8.69	100	11.50
Cameroon	19.21	210	10.93
Canada	1,240.06	10078	8.13
Chile	147.67	619	4.19
China	3,839.28	6049	1.58
Congo	15.67	57	3.64
Costa Rica	25.02	137	5.48
Côte d'Ivoire	18.17	90	4.95
Croatia	45.87	506	11.03
Cuba	55.44	177	3.19
Cyprus	19.21	85	4.43
Czech Republic	148.48	581	3.91
Denmark	256.82	2757	10.74
Ecuador	48.76	110	2.26
Egypt	121.04	459	3.79
Estonia	13.90	181	13.02
Ethiopia	20.40	235	11.52
Finland	204.15	1973	9.66
France	2,204.45	8777	3.98
Gabon	9.68	64	6.61
Gambia	0.78	73	93.08
Georgia	8.24	41	4.97
Germany	2,954.36	9021	3.05
Ghana	14.80	197	13.31
Greece	240.95	2029	8.42
Guatemala	32.56	72	2.21
Hungary	109.26	571	5.23
Iceland	16.39	259	15.80

Country	2010 GDP USDbn	No papers	No papers / GDP
India	1,243.68	2843	2.29
Indonesia	377.90	209	0.55
Iran	242.70	1326	5.46
Iraq	67.27	37	0.55
Ireland	203.31	955	4.70
Israel	169.01	1546	9.15
Italy	1,763.89	7480	4.24
Jamaica	11.08	90	8.13
Japan	4,648.47	4274	0.92
Jordan	17.03	132	7.75
Kazakhstan	77.25	33	0.43
Kenya	23.53	482	20.49
Kuwait	85.61	154	1.80
Laos	4.02	52	12.93
Latvia	15.50	49	3.16
Lebanon	30.75	183	5.95
Lithuania	27.35	187	6.84
Luxembourg	40.70	115	2.83
Macedonia	7.14	38	5.32
Madagascar	5.76	60	10.42
Malawi	3.29	143	43.46
Malaysia	178.67	445	2.49
Mali	6.97	72	10.33
Malta	6.65	47	7.06
Mexico	953.07	1445	1.52
Mongolia	3.45	47	13.61
Morocco	75.52	198	2.62
Mozambique	9.13	96	10.52
Nepal	10.10	148	14.65
Netherlands	683.06	5860	8.58
New Zealand	120.04	1361	11.34
Nicaragua	7.16	45	6.28
Niger	4.38	43	9.81
Nigeria	159.02	616	3.87
Norway	315.80	1993	6.31
Oman	41.94	53	1.26
Pakistan	129.52	462	3.57
Panama	22.60	36	1.59
Papua New Guinea	6.55	50	7.63
Paraguay	11.15	32	2.87
Peru	103.49	335	3.24
Philippines	131.13	235	1.79
Poland	383.21	1215	3.17

Country	2010 GDP USDbn	No papers	No papers / GDP
Portugal	197.16	898	4.55
Qatar	101.93	75	0.74
Republic of Korea	1,098.69	1796	1.63
Romania	114.09	415	3.64
Russia	909.24	656	0.72
Rwanda	3.79	41	10.83
Saudi Arabia	435.99	338	0.78
Senegal	10.37	159	15.34
Serbia	27.88	284	10.19
Singapore	176.46	827	4.69
Slovakia	76.90	272	3.54
Slovenia	39.03	287	7.35
South Africa	289.81	1579	5.45
Spain	1,179.23	6089	5.16
Sri Lanka	33.25	146	4.39
Sudan	35.82	92	2.57
Sweden	401.62	4084	10.17
Switzerland	427.58	3587	8.39
Syria	32.03	38	1.19
Tanzania	19.72	341	17.29
Thailand	210.09	958	4.56
Trinidad and Tobago	18.99	74	3.90
Tunisia	40.74	376	9.23
Turkey	565.09	1894	3.35
Uganda	13.36	326	24.40
Ukraine	90.58	77	0.85
United Arab Emirates	204.45	158	0.77
United Kingdom	2,360.03	18918	8.02
United States of America	13,595.64	59416	4.37
Uruguay	22.90	126	5.50
Uzbekistan	21.49	30	1.40
Venezuela	174.55	366	2.10
Viet Nam	78.28	316	4.04
Zambia	9.80	131	13.37
Zimbabwe	5.20	122	23.45

Appendix 5

Full results – h-index/GDP in 2006-2010

Country	2010 GDP USDbn	2006-2010 H index	H index/GDP
Afghanistan	10.24	11	1.07
Algeria	116.51	17	0.15
Angola	50.37	10	0.20
Argentina	293.70	55	0.19
Australia	797.44	143	0.18
Austria	325.55	70	0.22
Bangladesh	81.47	34	0.42
Barbados	4.03	10	2.48
Belgium	400.38	115	0.29
Benin	5.23	14	2.68
Bolivia	11.95	20	1.67
Botswana	12.12	14	1.16
Brazil	1,096.75	78	0.07
Bulgaria	32.99	26	0.79
Burkina Faso	7.11	25	3.52
Cambodia	8.69	23	2.65
Cameroon	19.21	30	1.56
Canada	1,240.06	171	0.14
Chile	147.67	39	0.26
China	3,839.28	100	0.03
Congo	15.67	17	1.09
Costa Rica	25.02	25	1.00
Côte d'Ivoire	18.17	25	1.38
Croatia	45.87	37	0.81
Cuba	55.44	24	0.43
Cyprus	19.21	17	0.89
Czech Republic	148.48	50	0.34
Denmark	256.82	108	0.42
Dominican Republic	47.85	11	0.23
Ecuador	48.76	22	0.45
Egypt	121.04	34	0.28
Estonia	13.90	33	2.37
Ethiopia	20.40	25	1.23
Fiji	3.03	12	3.96
Finland	204.15	95	0.47
France	2,204.45	152	0.07
Gabon	9.68	19	1.96
Gambia	0.78	22	28.05
Georgia	8.24	11	1.33
Germany	2,954.36	155	0.05
Ghana	14.80	27	1.82
Greece	240.95	85	0.35
Guatemala	32.56	18	0.55

Country	2010 GDP USDbn	2006-2010 H index	H index/GDP
Honduras	11.55	10	0.87
Hungary	109.26	53	0.49
Iceland	16.39	54	3.30
India	1,243.68	72	0.06
Indonesia	377.90	33	0.09
Iran	242.70	42	0.17
Ireland	203.31	71	0.35
Israel	169.01	73	0.43
Italy	1,763.89	146	0.08
Jamaica	11.08	11	0.99
Japan	4,648.47	95	0.02
Jordan	17.03	17	1.00
Kazakhstan	77.25	11	0.14
Kenya	23.53	52	2.21
Kuwait	85.61	21	0.25
Laos	4.02	18	4.48
Latvia	15.50	18	1.16
Lebanon	30.75	29	0.94
Lithuania	27.35	22	0.80
Luxembourg	40.70	27	0.66
Madagascar	5.76	18	3.13
Malawi	3.29	29	8.81
Malaysia	178.67	37	0.21
Mali	6.97	23	3.30
Malta	6.65	17	2.56
Mexico	953.07	59	0.06
Mongolia	3.45	11	3.18
Morocco	75.52	18	0.24
Mozambique	9.13	22	2.41
Nepal	10.10	23	2.28
Netherlands	683.06	157	0.23
New Zealand	120.04	70	0.58
Nicaragua	7.16	14	1.95
Niger	4.38	17	3.88
Nigeria	159.02	31	0.19
Norway	315.80	84	0.27
Oman	41.94	12	0.29
Pakistan	129.52	33	0.25
Panama	22.60	14	0.62
Papua New Guinea	6.55	17	2.59
Paraguay	11.15	15	1.35
Peru	103.49	35	0.34
Philippines	131.13	27	0.21

Country	2010 GDP USDbn	2006-2010 H index	H index/GDP
Poland	383.21	63	0.16
Portugal	197.16	56	0.28
Qatar	101.93	16	0.16
Republic of Korea	1,098.69	64	0.06
Romania	114.09	31	0.27
Russia	909.24	36	0.04
Rwanda	3.79	15	3.96
Saudi Arabia	435.99	26	0.06
Senegal	10.37	24	2.32
Serbia	27.88	22	0.79
Singapore	176.46	62	0.35
Slovakia	76.90	34	0.44
Slovenia	39.03	29	0.74
South Africa	289.81	77	0.27
Spain	1,179.23	123	0.10
Sri Lanka	33.25	23	0.69
Sudan	35.82	17	0.47
Sweden	401.62	123	0.31
Switzerland	427.58	143	0.33
Syria	32.03	12	0.37
Tanzania	19.72	36	1.83
Thailand	210.09	56	0.27
Trinidad and Tobago	18.99	11	0.58
Tunisia	40.74	24	0.59
Turkey	565.09	48	0.08
Uganda	13.36	39	2.92
Ukraine	90.58	19	0.21
United Arab Emirates	204.45	21	0.10
United Kingdom	2,360.03	223	0.09
United States of America	13,595.64	294	0.02
Uruguay	22.90	23	1.00
Venezuela	174.55	31	0.18
Viet Nam	78.28	34	0.43
Yemen	19.99	10	0.50
Zambia	9.80	22	2.24
Zimbabwe	5.20	23	4.42
